# Trabecular Bone Parameters, TIMP-2, MMP-8, MMP-13, VEGF Expression and Immunolocalization in Bone and Cartilage in Newborn Offspring Prenatally Exposed to Fumonisins

**DOI:** 10.3390/ijms222212528

**Published:** 2021-11-20

**Authors:** Ewa Tomaszewska, Halyna Rudyk, Izabela Świetlicka, Monika Hułas-Stasiak, Janine Donaldson, Marta Arczewska, Siemowit Muszyński, Piotr Dobrowolski, Maria Mielnik-Błaszczak, Marcin Bartłomiej Arciszewski, Volodymyr Kushnir, Oksana Brezvyn, Viktor Muzyka, Ihor Kotsyumbas

**Affiliations:** 1 Department of Animal Physiology, Faculty of Veterinary Medicine, University of Life Sciences in Lublin, Akademicka St. 12, 20-950 Lublin, Poland; 2 State Scientific Research Control Institute of Veterinary Medicinal Products and Feed Additives, Donetska St. 11, 79000 Lviv, Ukraine; galusik.77@gmail.com (H.R.); wolodjak@gmail.com (V.K.); brezvun@gmail.com (O.B.); muzyka@scivp.lviv.ua (V.M.); dir@scivp.lviv.ua (I.K.); 3 Department of Biophysics, Faculty of Environmental Biology, University of Life Sciences in Lublin, Akademicka St. 13, 20-950 Lublin, Poland; marta.arczewska@up.lublin.pl (M.A.); siemowit.muszynski@up.lublin.pl (S.M.); 4 Department of Functional Anatomy and Cytobiology, Faculty of Biology and Biotechnology, Maria Curie-Sklodowska University, 19 Akademicka St., 20-033 Lublin, Poland; monhul@o2.pl (M.H.-S.); piotr.dobrowolski@umcs.lublin.pl (P.D.); 5 School of Physiology, Faculty of Health Sciences, University of the Witwatersrand, 7 York Road, Parktown, Johannesburg 2193, South Africa; janine.donaldson@wits.ac.za; 6 Chair and Department of Developmental Dentistry, Medical University of Lublin, 7 Karmelicka St., 20-081 Lublin, Poland; maria.mielnik-blaszczak@umlub.pl; 7 Department of Animal Anatomy and Histology, University of Life Sciences in Lublin, 20-950 Lublin, Poland; mb.arciszewski@wp.pl

**Keywords:** fumonisins (FB), newborn rats, bone, articular cartilage, matrix metalloproteinase 13 (MMP-13), matrix metalloproteinase 8 (MMP-8), tissue inhibitor of metalloproteinases 2 (TIMP-2)

## Abstract

Fumonisins are protein serine/threonine phosphatase inhibitors and potent inhibitors of sphingosine N-acyltransferase (ceramide synthase) disrupting de novo sphingolipid biosynthesis. The experiment was conducted to evaluate the effects of fumonisins (FB) exposure from the 7th day of pregnancy to parturition on offspring bone development. The rats were randomly allocated to either a control group (*n* = 6), not treated with FBs, or to one of the two groups intoxicated with FBs (either at 60 mg FB/kg b.w. or at 90 mg FB/kg b.w. Numerous negative, offspring sex-dependent effects of maternal FB exposure were observed with regards to the histomorphometry of trabecular bone. These effects were due to FB-inducted alterations in bone metabolism, as indicated by changes in the expression of selected proteins involved in bone development: tissue inhibitor of metalloproteinases 2 (TIMP-2), matrix metalloproteinase 8 (MMP-8), matrix metalloproteinase 13 (MMP-13), and vascular endothelial growth factor (VEGF). The immunolocalization of MMPs and TIMP-2 was performed in trabecular and compact bone, as well as articular and growth plate cartilages. Based on the results, it can be concluded that the exposure of pregnant dams to FB negatively affected the expression of certain proteins responsible for bone matrix degradation in newborns prenatally exposed to FB in a dose- and sex-dependent manner.

## 1. Introduction

**Figure 1:**
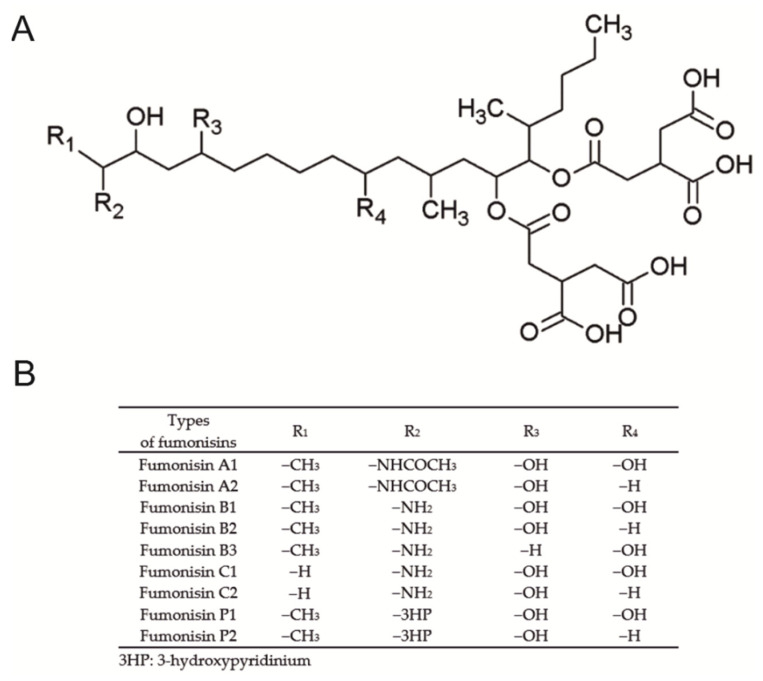
Chemical structure (**A**) and possible analogs of fumonisin (**B**).

Nutrition has a significant effect on both human and animal health. Improper preparation and storage of feed can result not only in significant economic losses, but also in the development of health problems in consumers. Cereal, commonly used in human and animal nutrition, regardless of the form of processing, in favorable climatic conditions, may be contaminated with fungi producing mycotoxins. The naturally occurring secondary products of the metabolism of *Fusarium* are fumonisins. There are various types of fumonisins, including types A, B, C and P, which like other mycotoxins are heat-resistant metabolites (Figure 1). 

Type B fumonisins (FB1 and FB2) occur frequently and are very toxic. Both types of FB are an inhibitor of sphingosine N-acyltransferase, but FB2 is a cytotoxic analog of FB1, which is the most abundant and additionally inhibits protein serine/threonine phosphatases [1]. Both FB1 and FB2, which were found in more than 50% of samples of feed and feed raw materials, interfere with sphingolipid metabolism [2,3]. Both types of FB have a similar toxicity and occur naturally in a ratio of FB1 to FB2 of about 3:1 [4]. The presence of FB1 and FB2 in human or animal feed is regulated by EU legislation [5,6]. The development of clinical signs of FB intoxication depends on the dose of FB to which one is exposed. The various clinical implications differ between animal species and are also dependent on the route of FB administration, as well as the sex and age of the animals [7]. Horses, pigs, sheep and rodents are more sensitive to FB compared to other animals and display non-species-specific symptoms, including hepatic or kidney toxicity [8,9,10,11], as well as some species-specific symptoms in target organs (such as the brain in horses and the lungs or esophagus in swine) [12]. The bioavailability and toxicity of FBs in ruminants and poultry is poor compared to that of other species. In poultry, morphological and functional changes following FB toxicity are dependent on avian species. Turkeys and ducks are more susceptible to FB toxicity than broiler chickens, in which doses of up to 300 mg/kg feed can induce clinical toxicity [12]. Fumonisin administration, even at a dose of 1.0 mg/kg b.w., leads to degradation of the intestinal barrier [13]. FBs have been shown to be hepatocarcinogenic in rats and mice [14]. They are also potentially hazardous to humans as a causative factor of immunosuppression or neurotoxicity [15,16]. In humans FBs are associated with stunted growth in children, and interference with folic acid uptake and thus are associated with folic acid deficiency-linked birth defects [17,18]. The International Agency for Research on Cancer (IARC) has designated FB1 in Group 2B meaning “possibly carcinogenic to humans” [19]. The European Commission and the FDA in the USA have issued guidelines on the total amount of fumonisins that are acceptable in human foods and animal feed [6,20]. FBs are most frequently detected in South American (77%), African (72%) and Southern European (70%) feed samples [21]. FBs have been detected in commodities such as maize, and in dried figs, and some plants [22]. Worldwide, human exposure to fumonisin B1 through maize is estimated to range from 0.01 to 354.0 μg/kg body weight [23].

The economic losses in the provision of healthcare or animal husbandry are difficult to calculate, since information concerning the subclinical effects of FBs on human or animal health, and animal productivity losses caused by chronic low level exposure, is limited [11,21,24]. More recently the fungus *Aspergillus niger* has also been shown to produce some fumonisin metabolites, which have been found in grapes, raisins, wine, and coffee [19].

As previously mentioned, FBs are present in moldy food intended for animals or humans including infants and young children or pregnant women. The involuntary intake of such products poses a health hazard. Thus, nutrition also plays an important role in prenatal development and has long-term effects that are evident later in life [25,26]. Taking into account the harmful influence of FBs on many body systems following exposure, it seems reasonable to investigate the effects of FB exposure during pregnancy on offspring bone development and expression and immunolocalization of selected proteins involved in bone development.

Therefore, the objective of the current study was to determine the effects of maternal FB supplementation on the histomorphometry of trabecular bone, as well as the immunolocalization of tissue inhibitor of metalloproteinases 2 (TIMP-2), matrix metalloproteinases (MMPs) in the bone tissue of newborn rats. In doing so, the present study examined (i) the changes in immunolocalization of MMP-8, MMP-13 and TIMP-2 exerted by FB supplementation during pregnancy in the rats, (ii) the detection of VEGF, TIMP-2 and MMP-8 by Western blot, and (iii) basal histomorphometrical parameters of bone trabeculae. Using standard immunohistochemistry methods, immunoexpression of the selected proteins was established in articular cartilage, growth plate cartilage, trabecular and compact bone. Together, these measurements should provide some fundamental information regarding outcomes of FB exposure during pregnancy on bone development of the offspring.

## 2. Results

### 2.1. Histomorphometry

A decrease in bone volume over the total volume (BV/TV), trabecular thickness (Tb.Th), maximum trabecular thickness (Tb.Th_max_) and bone length was observed in females after maternal FB administration (Table 1). The observed decrease in the abovementioned bone parameters was not confirmed to be dose-related; however, linear and quadratic trends proved to be statistically significant for all the variables. Quadratic trends were observed in both trabecular space (Tb.Sp) and trabecular number (Tb.N). A dose of 60 mg of FBs resulted in an increase in Tb.Sp, while FB at a dose of 90 mg resulted in a decrease in Tb.Sp. The opposite effect was observed in the case of Tb.N, where the 60 mg dose caused a decrease in Tb.N and the 90 mg dose caused an increase. The maximum trabecular space was unchanged, irrespective of FB dose, compared to the control group (*p* = 0.259).

Histomorphometric results for males (Table 1) revealed that FB intoxication did not influence BV/TV, Tb.Th, Tb.Th_max_ and Tb.N, irrespective of dose. Tb.Sp increased substantially following FB intoxication at a dose of 60 mg; however, the 90 mg dose had no significant effect. Maximum Tb.Sp also increased following the 60 mg dose and decreased following the 90 mg dose, with the differences between doses being statistically significant. For both Tb.Sp and Tb.Sp_max_, a significant quadratic trend was observed. Similar to that observed in the females, the bones in the males were significantly shortened under the influence of fumonisin, but no differences in length between the doses were observed. Both linear and quadratic trends were detected.

### 2.2. Immunostaining

**Figure 2:**
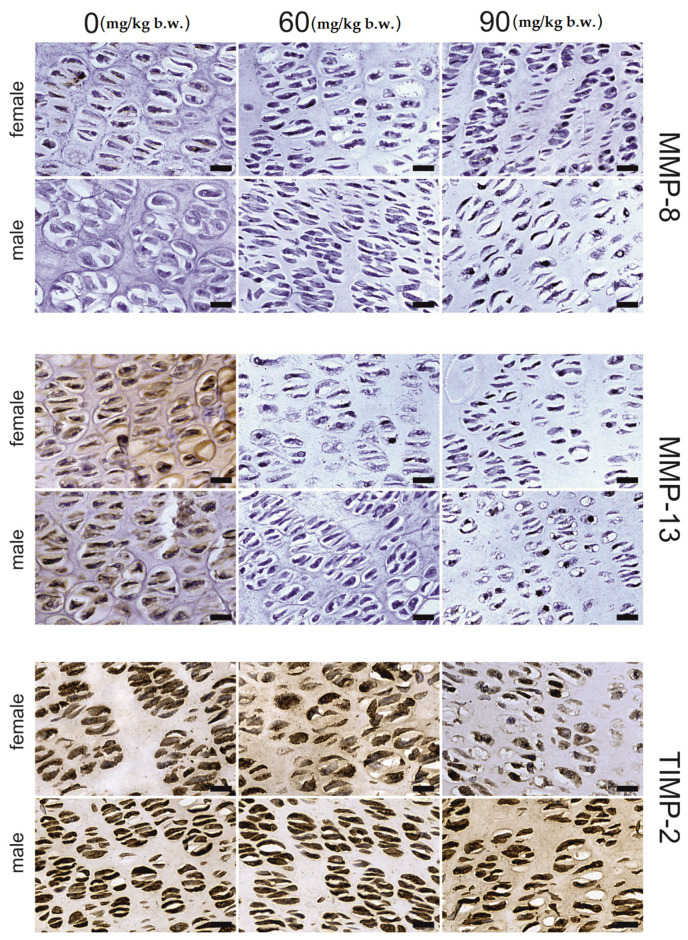
Representative images of the immunohistochemical reactions for MMP-8, MMP-13 and TIMP-2 from the proliferative zone of femoral growth plate of newborn rats (female and male) exposed prenatally to 0, 60 and 90 mg/kg b.w. of fumonisins. All the scale bars represent 20 µm.

**Figure 3:**
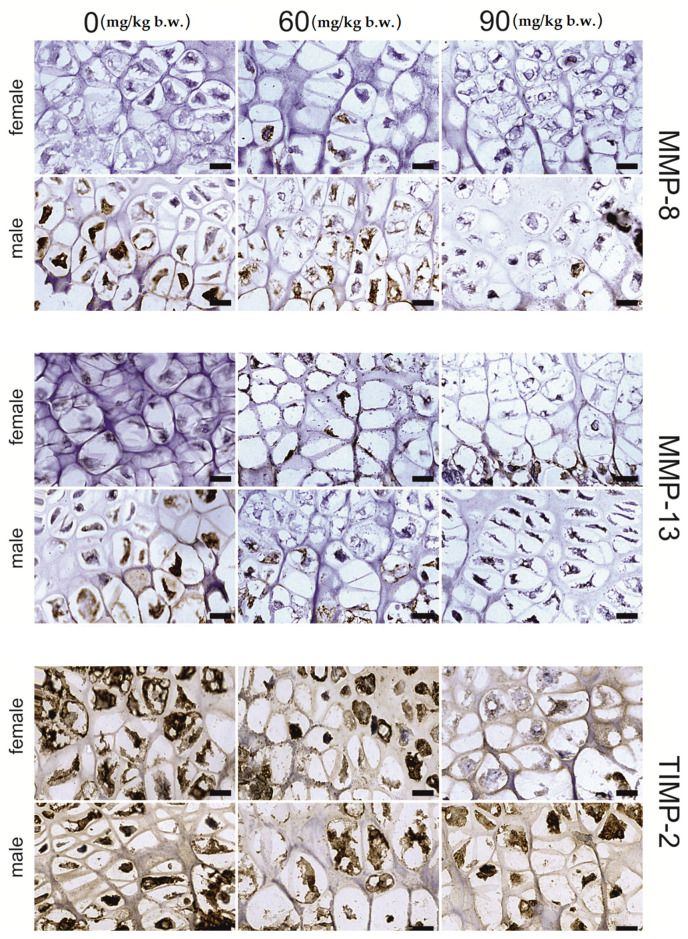
Representative images of the immunohistochemical reactions for MMP-8, MMP-13 and TIMP-2 from the hypertrophic zone of the femoral growth plate of newborn rats (female and male) exposed prenatally to 0, 60 and 90 mg/kg b.w. of fumonisins. All the scale bars represent 20 µm.

The growing plate of female rats, both in the proliferative and hypertrophic regions, was prone to FB intoxication (Table 2). The intensity of MMP-13, MMP-8 and TIMP-2 immunostaining of the MMP-13, MMP-8 and TIMP-2 proteins, was region- and dose-dependent. A general decrease in the intensity of the immunohistochemical reaction for the analyzed proteins was observed in the proliferative zone (Figure 2), apart from that of MMP-13, which showed a decrease in reaction intensity following the 60 mg FB dose. On the other hand, following the 90 mg dose of FB, an increase in reaction intensity was observed. Furthermore, the strong linear and quadratic effects observed for all the proteins assessed suggest that the increase in FB dose might cause linear or quadratic changes in protein expression. In the hypertrophic zone (Figure 3), the 90 mg FB dose caused a significant increase (*p* = 0.005) in MMP-13, while the mean amount of MMP-8 was not different (*p* = 0.188) between groups. In turn, TIMP-2 expression decreased following FB exposure, and both linear and quadratic trends proved to be statistically significant (*p* < 0.001).

**Figure 4:**
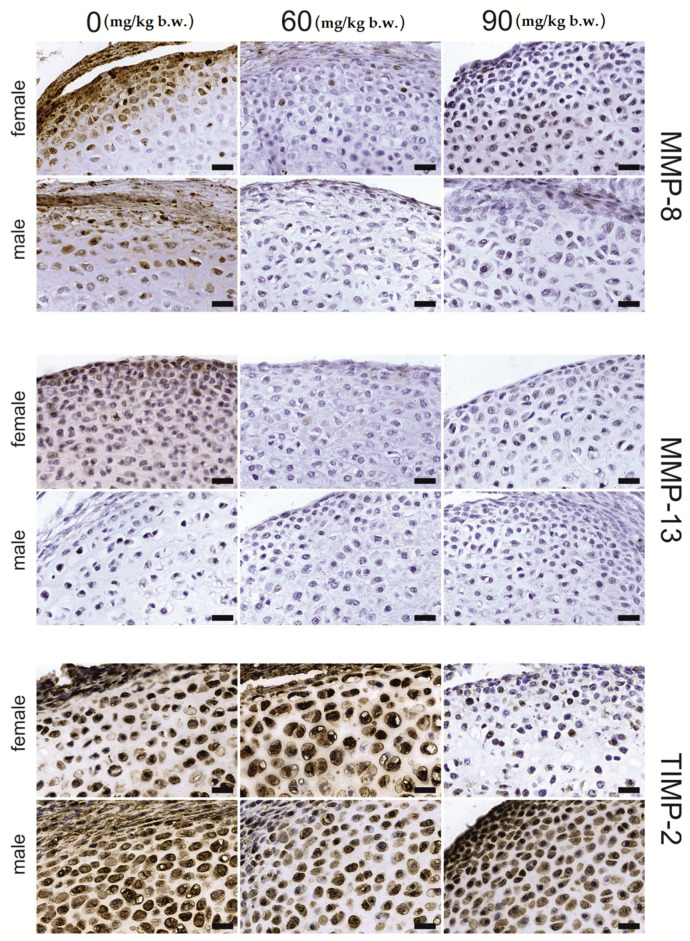
Representative images of the immunohistochemical reactions for MMP-8, MMP-13 and TIMP-2 from the articular cartilage of newborn rats (female and male) exposed prenatally to 0, 60 and 90 mg/kg b.w. of fumonisins. All the scale bars represent 40 µm.

The articular cartilage tissue responded to FB intoxication with a significant decrease in the intensity of MMP-13 (*p* < 0.001) and MMP-8 (*p* < 0.001) protein staining when compared to the control group; however, no statistical differences between FB doses were detected. For both proteins, linear and quadratic trends were observed (*p* < 0.001). Additionally, no changes in TIMP-2 immunostaining expression were observed (Figure 4). 

**Figure 5:**
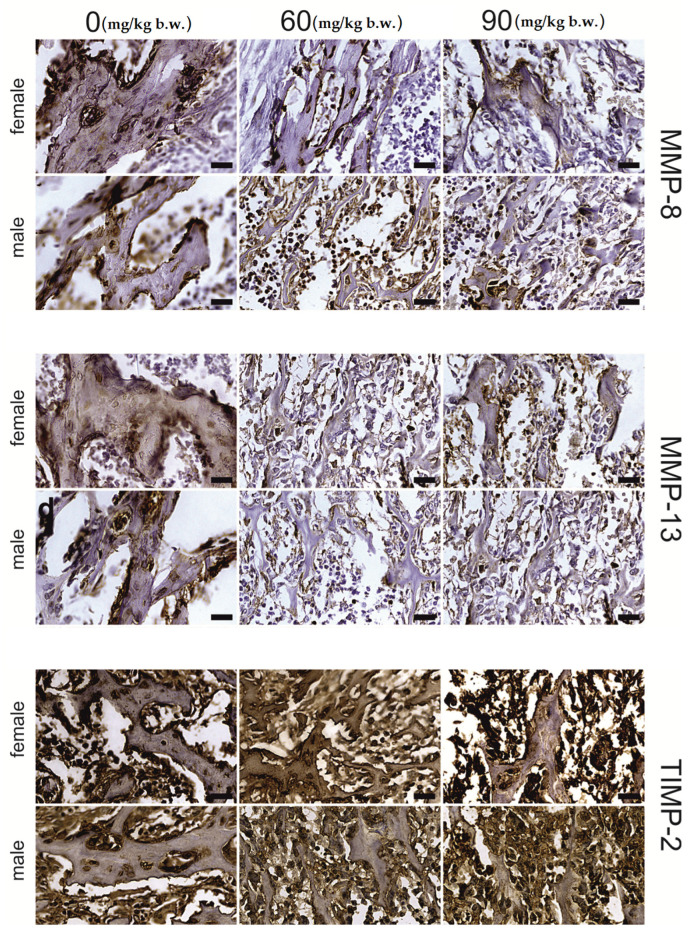
Representative images of the immunohistochemical reactions for MMP-8, MMP-13 and TIMP-2 from the trabecular bone of newborn rats (female and male) exposed prenatally to 0, 60 and 90 mg/kg b.w. of fumonisins. All the scale bars represent 40 µm. Sample pictures of the trabecular bone with signed trabeculae, osteocytes and marrow space are presented in the Supplementary Materials, Figure S2.

For the osteocytes in the trabecular bone, a significant decline in the immunosignal of MMP-13 (*p* = 0.001) and MMP-8 (*p* < 0.001) proteins, with statistically significant linear and quadratic trends, was observed. Furthermore, a similar effect was observed with regards to MMP-13 expression in the matrix, while MMP-8 expression showed a strong quadratic trend (*p* = 0.002)—decreasing following the 60 mg FB dose and increasing following the 90 mg FB dose. No significant changes in the immunostaining for TIMP-2 protein were observed after FB exposure for both the osteocytes and the matrix (Figure 5).

FB exposure had a significant effect on the expression of the proteins in the osteocytes (*p* < 0.001). However, it should be noted that this relationship was somewhat ambiguous, suggesting the existence of a strong linear downward trend for MMP-13, MMP-8 and TIMP-2 proteins and a quadratic trend for MMP-8 and TIMP-2. In the case of the matrix, no changes in the intensity of the immunostaining reaction of the proteins assessed were recorded. 

For males (Table 3), MMP-13 and MMP-8 staining (Figure 2 and Figure 3) were not different in the growing plate following FB exposure (*p* = 0.771 and *p* = 0.071, respectively). In contrast, statistically significant changes in the means (*p* < 0.001) were recorded for TIMP-2 in the proliferative and hypertrophic regions. Furthermore, for those regions, significant linear and quadratic trends were observed (*p* < 0.001). 

The articular tissue of male rats responded to FB exposure with a significant (*p* < 0.001) decrease in the immunosignal of MMP-13 and MMP-8 compared to that observed in the control group, with no differences observed between the two FB doses (Figure 4). Significant linear and quadratic trends were observed for the proteins mentioned. On the other hand, TIMP-2 staining was significantly decreased following the 60 mg FB dose compared to that observed in the control group, while the 90 mg FB dose significantly increased the intensity of staining of this protein. No significant differences in immunostaining were observed between the control and 60 mg FB dose groups. Therefore, it is important to underline a statistically significant (*p* = 0.003) quadratic effect in this case. 

The immunohistochemical staining reaction for MMP-13, MMP-8 and TIMP-2 in trabecular bone osteocytes (Figure 5) significantly decreased following both FB doses compared to that of the control group (*p* < 0.001). In addition, a significant inversely proportional linear relationship between the dose and the reaction intensity for all examined proteins was confirmed (*p* < 0.001). 

A significant quadratic effect was observed with regards to both MMP-8 and TIMP-2 immunostaining (*p* < 0.001). The matrix of the trabecular bone showed significantly lower staining of MMP-13 and TIMP-2 (*p* < 0.001) following increasing doses of FB. However, the differences between FB doses were only significant in the case of TIMP-2 (*p* < 0.001). There were no significant differences in MMP-8 staining between the control and experimental groups (*p* = 0.114). Despite the lack of differences, a linear effect was observed at the limit of significance (*p* = 0.048).

**Figure 6:**
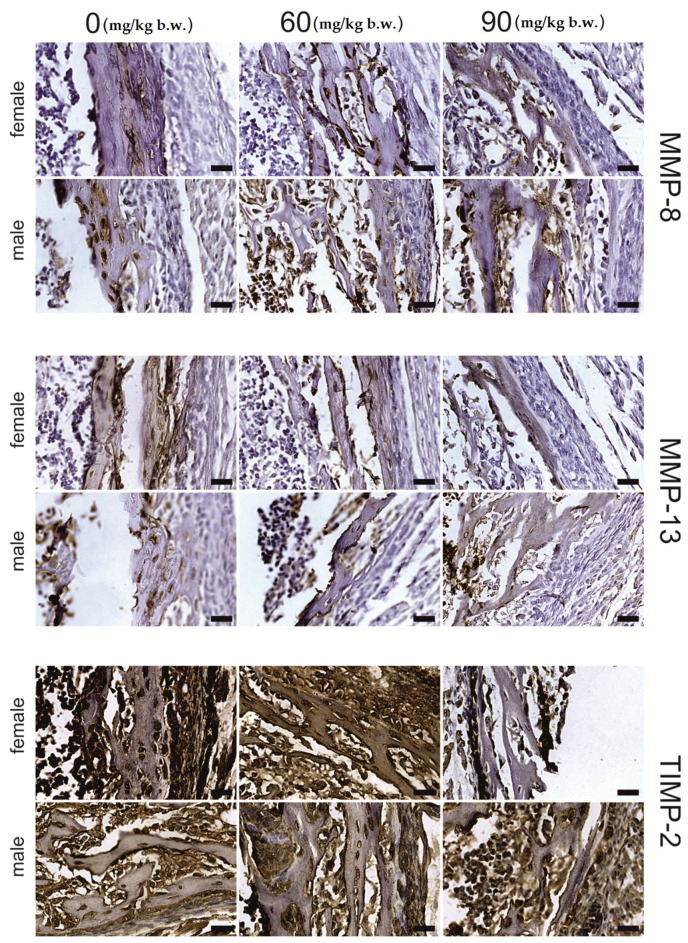
Representative images of the immunohistochemical reactions for MMP-8, MMP-13 and TIMP-2 from the compact bone of newborn rats (female and male) exposed prenatally to 0, 60 and 90 mg/kg b.w. of fumonisins. All the scale bars represent 40 µm. Sample pictures of the compact bone with signed periosteum, osteocytes and bone marrow cavity are presented in the Supplementary Materials, Figure S3.

Osteocytes of compact bone (Figure 6) showed significant differences in the staining of MMP-13 and MMP-8 under the influence of FB (*p* < 0.001). The immunosignal for MMP-13 was lower following both FB doses compared to that of the control group. Significant linear and quadratic effects were also observed (*p* < 0.001). The MMP-8 signal was decreased following the 60 mg FB dose, and increased following the 90 mg dose. Additionally, a significant quadratic trend (*p* < 0.001) for MMP-8 was observed. MMP-13 staining in the compact bone matrix was significantly increased, by about 30%, compared to that of the control group, following the 60 mg FB dose. The 90 mg FB dose caused a decrease in MMP-13 staining to a value which was not different to that of the control group. The contrast analysis proved the quadratic trend (*p* = 0.001) for MMP-13 immunostaining. The opposite effect was observed for MMP-8, where the 60 mg FB dose caused a significant decrease in the intensity of the reaction. The 90 mg FB dose induced more than a twofold increase in the expression of MMP-8. The linear trend proved to be statistically significant (*p* = 0.002). FB exposure did not affect TIMP-2 staining in compact bone (*p* = 0.679).

### 2.3. Western Blot Protein Expression

**Figure 7:**
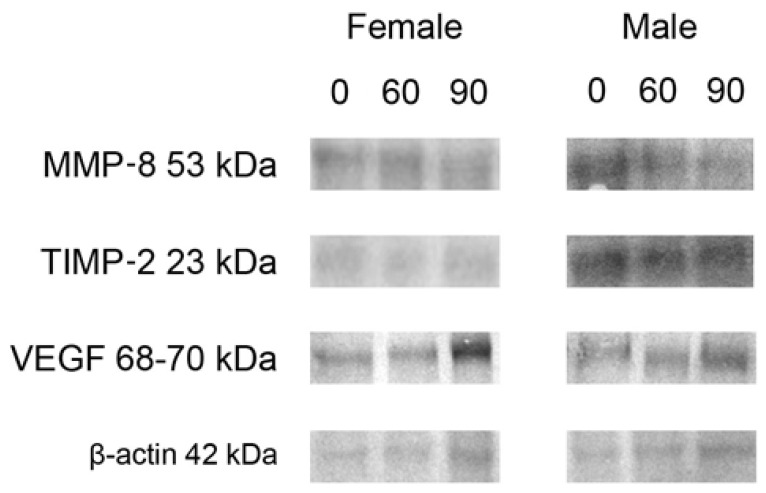
Representative Western blots of MMP-8, TIMP-2 and VEGF as well as β-actin as internal control in femur samples obtained from the newborns prenatally exposed to 0, 60 and 90 mg FB/kg b.w. The relative values of abundance of MMP-8, TIMP-2 and VEGF expressed as normalized ratio to housekeeping protein β-actin used as a loading control with results of corresponding statistical analysis are presented in Table 4. Original Western blot membranes are presented in Supplementary Materials, Figure S4.

For both sexes, Western blot analysis showed that the expression of MMP-8 protein decreased significantly following the 90 mg FB dose, compared to the control group (Figure 7: representative Western blots; Table 4: protein levels presented as normalized ratio to β-actin). No significant changes in MMP-8 protein expression were observed following the 60 mg FB dose. Although the level of TIMP-2 proteins in females increased following the 60 mg FB dose and decreased following the 90 mg dose, a statistically significant difference was only detected between the doses considered. 

There was no change in TIMP-2 expression in males. An increase in VEGF protein levels was observed in females, with the effect of the 90 mg FB dose being stronger than that of the lower FB dose. The analysis of trends showed that both linear and quadratic ones were statistically significant. The 60 mg FB dose caused a considerable increase in VEGF level in male rats, while the 90 mg FB dose returned VEGF protein expression to a value comparable to that of the control group. The abovementioned changes indicate the possibility of a quadratic trend, which proved to be statistically significant.

## 3. Discussion

The toxic effect exerted by FBs involves their inhibitory action on ceramide synthases, enzymes which play an essential role in sphingolipid metabolism. FB toxicity affects many signaling systems involved in cellular growth and differentiation. FBs have been proven to reduce weight gain and feed efficiency [27]. Chronic exposure to FBs induces intestinal damage, including to the enteric nervous system. FB exposure is toxic to both the duodenum and jejunum, where significant changes in morphology or cell proliferation have been observed [10,28]. To date, numerous studies which have investigated the prenatal effects of FB have observed neural tube defects following FB intoxication, which appear to be associated with mycotoxin, genetic, epigenetic, and metabolic factors, in a dose-dependent manner [29]. They have been observed in many dose-response studies in rats and mice, where the dose of FB was 10, 25, 55, 100, 125 or 150 mg FB/kg b.w. [30,31,32]. Animal studies have highlighted various other effects of mycotoxins including intensified endosteal resorption, thinning of the bone wall and overall weakening of the whole bone [33,34]. However, no previous studies have looked at the effects of prenatal FB exposure on the development of the skeletal system. 

The toxicity of fumonisins is based on the disturbance of sphingolipid synthesis. FB1 blocks biosynthesis of de novo sphingolipids through ceramide synthase inhibition. Sphingolipids are lipid components of each cell’s phospholipid bilayer membrane. There are simple and complex sphingolipids. The group of complex sphingolipids includes sphingomyelins, cerebroides, sulphatides and gangliosides. Sphingomyelin is an abundant member of the sphingolipid family whose metabolism plays a central role in ceramide biology. Ceramides, which are synthetized from serine and palmitoyl CoA in the endoplasmic reticulum, induce apoptosis, regulate cell differentiation, immunity, and participate in inflammatory responses [35]. As an intracellular lipid mediator, ceramides can regulate chondrocyte behavior [36]. It is known that abnormal lipid metabolism has a potential role in bone marrow lesions, synovial inflammation, and also osteoarthritic changes in chondrocytes [36]. Chondrocytes are responsible for preserving articular cartilage integrity by formation and maintaining the extracellular matrix, and a reduction of cartilage cellularity is a hallmark of osteoarthritis [37]. Sphingolipids are key mediators involved in the regulation of the physiological response of articular chondrocytes to harmful factors, which modulate signal transduction. These regulatory mechanisms of sphingolipids are important for maintaining cartilage homeostasis. Lost ECM (the extracellular matrix) homeostasis is a central event in pathogenesis of osteoarthritis that results from increased expression and activity of proteolytic enzymes degrading ECM, and decreased expression of ECM proteins, such as aggrecan or collagen type 2 [37]. This disturbance in the lipid metabolism of chondrocytes is linked with pro-inflammatory events in osteoarthritis and in turn the biosynthesis of key players such as VEGF, MMP-13 and MMP-8, which regulate bone formation [37,38]. Moreover, ceramides play an intricate role in the Golgi apparatus, and being responsible for ceramide-mediated cell death, they are responsible for how chondrocytes deal with stress and how the chondrocyte responds during skeletal development [35,39]. BMP signaling via the Akt cell signaling pathway (phosphatidylinositol 3-kinase and protein kinase B) is required for normal hypertrophic cell maturation and endochondral bone growth during cartilage development, and is also implicated in the regulation of lipid metabolism in chondrocytes [40]. The Akt pathway regulates many processes including metabolism (Akt enhances protein synthesis through increasing the phosphorylation of the mammalian target of rapamycin), proliferation, cell survival, growth and angiogenesis, and takes part in BMP-induced chondrogenesis by the promotion of chondrocyte differentiation; while ceramides inhibits this pathway by the reduction of the level of Akt phosphorylation and the activation of protein phosphatase 2A [40]. Some proinflammatory cytokines that are widely implicated in the pathogenesis of arthritis increase ceramides through hydrolysis of the cell membrane lipid sphingomyelin by endosomal acidic and membrane-bound neutral sphingomyelinases [40]. In turn, ceramides also play a role in the differentiation of chondroprogenitor cell populations promoting the chondrogenic differentiation of bone marrow mesenchymal stem cells [41]. Disruption in glycosphingolipid synthesis enhances the development of osteoarthritis in mice [42]. Studies have also examined roles for ceramides in the regulation of type II collagen synthesis via the ERK (extracellular-signal-regulated kinase) signaling pathway [43], proteoglycans and glycosaminoglycans [44,45,46] and MMPs by chondrocytes [47].

Metabolically active bone tissue undergoes various processes throughout its lifetime. Before reaching peak bone mass, the predominant process is osteogenesis. In animals, the time by which the whole skeleton becomes completely ossified is not exactly known. In humans, by 25 years of age nearly all the bones are completely ossified. During this time there are two main processes that take place, bone formation and bone resorption. These processes together constitute bone remodeling. After reaching peak bone mass, the process of bone remodeling slows down, and a gradual loss of bone mass starts [48,49,50,51]. Bone tissue remodeling, irrespective of sex, is strictly controlled by many different factors including hormones, vitamins, cytokines, growth factors, nutrition and even harmful factors, which influence the activity of osteoblasts and osteoclasts, as well as chondrocytes and their precursors. The ECM is also a dynamic network of many interacting macromolecules including collagens, fibronectin, laminin and proteoglycans, which are degraded by endopeptidases (collagenases or gelatinases), including metalloproteinases. Since the ECM is subject to dynamic changes all the time, its structure is preserved thanks to the balance of interactions between MMPs, which are responsible for the degradation of ECM proteins (mainly collagen and elastin), and their endogenous tissue inhibitors, such as TIMP [52]. 

The expression of TIMP-2, a natural inhibitor of the matrix metalloproteinases, was decreased following FB exposure in a dose- and sex-responsive manner. The significant decrease in TIMP-2 observed in the female newborn rats of the current study was mainly observed in the growth plate, as well as in the compact bone, although it was not supported fully by WB analysis, which showed that FBs did not influence TIMP-2 expression in FB exposed animals compared to the control group. On the other hand, in the case of the 90 mg FB/kg b.w. dose, WB analysis indicated a decrease in TIMP-2 expression compared to the 60 mg FB/kg b.w. dose in females. Furthermore, TIMP-2 expression in male newborns was observed in the growth plate and trabecular bone (in both cells and matrix), with a dose-dependent reduced TIMP-2 expression in articular cartilage. However, the WB analysis did not support the results noted in males. The WB analysis was performed on the whole bone, including the bone marrow. It should be emphasized that TIMP-2 or its RNA is detected in all tissues. The hematopoietic cells in bone marrow secrete many growth factors (including TIMP-2) and produce numerous extracellular matrix proteins [53]. This could be the main reason why our WB analysis did not produce the same or similar results as that of the immunostaining, where each bone compartment was analyzed separately. Additionally, TIMP-2 expression was very strong in cells from the bone marrow of our newborn rats, irrespective of sex. The cell growth-promoting activity of TIMP-2 is concentration-dependent and includes anti-angiogenic activity, which in turn involves MMP-inhibition [52]. The dose and compartment-dependent changes in TIMP-2 expression observed in the current study indicated that the differences in bone matrix turnover processes were influenced by the sex of the newborn rats. TIMP-2 is also responsible for the stimulation of osteoclastic bone resorption [54,55]. FB exposure led to decreased TIMP-2 expression in the male newborn rats of the current study, which was associated with very few changes in trabecular bone. A dose-dependent increase in trabecular separation was observed, indicating that both FB doses used in the current study shortened the bone length, but only the lower FB dose exerted harmful effects on the trabecular bone parameters assessed. The opposite effect was observed in the female rats, where both FB doses negatively influenced the trabecular bone parameters and shortened the bone length. 

There is a dearth of studies which investigate the effects of FB exposure on bone metabolism, and no previous studies, to our knowledge, have investigated the effects of prenatal FB exposure on offspring bone metabolism. However, the available literature indicates that FBs are etiological factors of leg deformities in livestock animals, which disturb mineral homeostasis and decrease mechanical bone endurance. Previous results also indicate that FBs affect cell proliferation in the growth plate, as well as the synthesis of proteoglycans in articular cartilage [10,56]. A previous study performed on minks intoxicated with deoxynivalenol has shown that mycotoxins do not alter bone mineral density, but they do shorten bone and intensify the process of endosteal resorption, leading to a thinner bone wall [33,34].

Longitudinal bone growth is also dependent on blood supply. The formation of new blood vessels depends on pro-angiogenic factors and their endogenous inhibitors, which influence the progression of pathological events. TIMP-2 is a natural inhibitor of angiogenesis. Vascular endothelial growth factor (VEGF) is a pro-angiogenic factor which promotes the expression of MMP to remodel the ECM in order to facilitate the invasion of new blood vessels. VEGF induces endothelial cell proliferation, promotes cell migration, inhibits apoptosis and induces permeabilization of blood vessels [57]. VEGF activates a number of different intracellular signaling pathways. Its effects on cell survival are mediated by the Flk1/VEGFR2-PI3K-Akt pathway [58].

The current study showed that prenatal FB exposure also influenced VEGF expression. The changes in VEGF expression probably interfered with the altered TIMP-2 expression and thus could have a significant effect on the formation of new blood vessels and supply of nutrients to the bone tissue, leading ultimately to disturbed bone growth. This possibility should be further investigated.

The maintenance of proper bone and matrix turnover processes is important for the correct formation, development and longitudinal growth of the bone. Normally, bone and cartilage resorption are dependent on the maintenance of a proper balance between MMPs and TIMP-2. The MMPs group of proteins plays an important role in the resorption process of tissue matrix components, which is necessary for multiple physiological processes including embryo development, bone remodeling, angiogenesis and tissue repair [59]. Many different proteins belong to the MMPs group and are classified according to their structure and function. The lack of certain proteins, e.g., MMP-13 (collagenase 3) during embryonic development, has a negative effect on the formation of growth plates, which exerts harmful effects on bone development and growth [60], while MMP-8 (collagenase 2 or neutrophil collagenase) is necessary for the regulation of the inflammatory process. MMP-8 is highly active during the progressive loss of connective tissue [61]. Both MMP-13 and MMP-8 also serve as enzymes, the primary function of which is the degradation of type I, II, III and IV collagens in the matrix during development and remodeling of bone and cartilage. These MMPs are secreted by many of the correctly developing cells including mast cells, osteoblasts, macrophages, lymphocytes and monocytes [62]. The TIMP family consists of four proteins that differ in terms of expression, regulation and interaction. These TIMP proteins interact with a given metalloproteinase which is found in the tissue matrix, in a latent form [63].

The current study was therefore performed to investigate the influence of prenatal FB exposure on the expression of MMPs, specifically MMP-13 and MMP-8, as well as on the TIMP-2 inhibitor in bone and cartilage tissue of newborn male and female rats. Both MMPs reported on in the current study remodel collagen present in the bone extracellular matrix, and participate in the degradation and regeneration of the ECM of surrounding chondrocytes or osteocytes [62]. The results obtained showed that MMP-13 expression is decreased by prenatal FB exposure, and is dependent on the region assessed, as well as the sex of the rats, and in the case of the compact bone its expression is also dependent on FB dose. Moreover, prenatal FB exposure significantly decreased MMP-8 expression. This effect was supported by the WB analysis, which showed a decrease in MMP-8 protein in both males and females prenatally exposed to the 90 mg FB/kg b.w. dose.

MMP-8 is responsible for the increase in cytokines observed during inflammatory processes and MMP-13 participates in osteoarthritis, which is initiated by an inflammatory process [61]. MMPs play the same role in articular cartilage as they do in bone tissue. Articular cartilage homeostasis depends on the balance between collagens, gelatins, matrix glycoproteins and proteoglycans. An imbalance in these factors leads to the loss of structural integrity in articular cartilage, which besides the natural lack of blood and lymph supply, and its aneural character, can lead to the degradation observed in osteoarthritis [61]. In general, FB exposure results in a decrease in the expression all the proteins in a dose- and sex-dependent manner. This can be a direct and indirect FB effect. A direct effect arises when FB crosses the placenta and depends on differences in the basal morphology of the placenta. This varies with species and the stage of placental maturity [64]. Indirect FB effects result from prenatal adaptation of the infective organism to changing conditions in the intrauterine environment [26].

The current study has some limitations: (i) this study lacks biochemical or hormonal blood analysis, analysis of biomarkers of bone turnover and basal blood morphology; and (ii) quantitative protein analysis. This study has numerous strengths: (i) to the best of our knowledge this is the first study that has examined the expression of key proteins responsible for bone matrix homeostasis in newborns prenatally exposed to FBs; (ii) results for both rat sexes are presented, as well as for different bone compartments; and (iii) WB analysis was performed for some of the proteins. The possible protein–protein interaction network between examined proteins (MMP-8, MMP-13, TIMP-2, VEGF) and lipid biosynthesis-related proteins inhibited by FB (ceramide synthase and serine/threonine phosphatase) are presented in Supplementary Materials, Figure S5. However, we still do not know if the presented effect is mainly due to a direct or indirect effect of FB intoxication. The FB prenatal influence on bone development should be further studied, especially the direct FB effect based on the link between lipid metabolism, sphingosine kinase and serine/threonine-protein kinase, MMPs, TIMP and VEGF.

## 4. Materials and Methods

The experiment was performed in accordance with EU Directive 2010/63/EU under the license of the State Scientific Research Control Institute of Veterinary Medicinal Products and Feed Additives in Lviv, Ukraine.

### 4.1. Animals and Experimental Design

Eighteen pregnant (5-weeks-old), Wistar rat dams were housed individually in polypropylene cages (the dimensions of 380 × 200 × 590 mm) and allowed a one-week acclimatization period, during which they were accustomed to the laboratory conditions. The dams were kept at a temperature of 21 ± 3 °C, humidity of 55 ± 5%, with a 12h/12h day/night cycle and had free access to water. After the acclimatization period, the rats were randomly allocated to either a control group (C group; *n* = 6), not treated with FBs or to one of the two groups intoxicated with FBs, either at 60 mg FB/kg b.w. or 90 mg FB/kg b.w. (each group consisted of 6 dams). The pregnant dams were fed a standard laboratory rodents diet ad libitum. Fumonisins were given by daily intragastric administration (75% FB1 and 25% FB2, respectively). FBs were given in 0.5 mL of 0.9% saline solution from the 7th day of the pregnancy to parturition by oral gavage, as previously described [10]. The FBs’ preparation is also described in a previous publication [13], where the concentration of stock FB solution was determined using an ELISA (Ridascreen Fumonisin, #R3401, detection limit: 25 ug/kg; R-Biopharm AG, Darmstadt, Germany), according to the manufacturer’s protocol. Control animals received saline solution in the corresponding amount and manner. The 90 mg FB/kg b.w. dose was equal to 0.1 of the established LD50 value, which is sufficient to induce subclinical intoxication when given to adolescent rats for 21 days [10,28]. The 60 mg dose was higher than that found to trigger embryonic neural tube defects when given during early pregnancy, and was equal to 0.15 of the established LD50 value (induced subclinical intoxication when given to adolescent rats for 21 days) [10,64]. No changes in pregnant dam behavior or basal health state were noted during assessment by a veterinarian. Daily weight gain, food and water intake did not differ between the groups of dams irrespective of the treatment they received. On the day of delivery, newborns from each group (*n* = 6 males; *n* = 6 females) were weighed and euthanized by CO_2_ inhalation, and they were then subjected to further analysis. 

After euthanasia, both femora were dissected from the newborn rats and the remaining soft tissues were removed using a scalpel blade. Right femora, after measuring for length, were fixed in phosphate-buffered 4% (*v*/*v*) paraformaldehyde (pH 7.0), while left femora were immediately frozen in liquid nitrogen and stored at −80 °C until they were subjected to Western blot analysis.

### 4.2. Histomorphometry

The site and size of the areas of interest that were analyzed were chosen on the basis of motoric properties of the adult rat body, the knee joint in particular, and are shown in Supplementary Materials, Figure S1. These selected sites are most laden by the weight of the rat’s body. After fixation for 48 h, bones were dehydrated through an ascending ethanol series (30–70%, *v*/*v*), fixed with nonpolar Ottix Plus and Ottix Sharper solvents (DiaPath, Martinengo, Italy) and embedded in paraffin blocks. Four-μm-thick sagittal sections of the middle part of the lateral condyle, separated by 10 μm between each section, were obtained using a microtome (HM360, Microm, Walldorf, Germany) [65]. The cuts were always carried out in the same orientation and plane, and the same regions are presented in all the pictures. The sections were stained with Goldner’s trichrome and then photographed in brightfield light, using a CX43 microscope (Olympus, Tokyo, Japan), equipped with a UC50 digital camera (Olympus, Tokyo, Japan) to evaluate basal morphology of trabeculae. For all analyses, three slides/bone with 20 μm separation were analyzed. The images were analyzed using ImageJ and CellSens (Olympus, Tokyo, Japan) image analysis software [66].

### 4.3. Immunohistochemistry

Immunohistochemical staining was performed according to the manufacturer’s protocols (Abcam, Cambridge, UK). For immunohistochemistry, rabbit monoclonal to matrix metallopeptidase 8 (MMP-8; ab81286), rabbit polyclonal to matrix metalloproteinases 18 (MMP-13; ab75606) and mouse monoclonal to tissue inhibitor of metalloproteinases 2 (TIMP-2; ab1828) antibodies, diluted in Diamond antibody diluent (Cell Marque Corp., Rocklin, CA, USA), were used as primary antibodies. Ready-to-use Bright Vision +Poly-HRP-Anti Ms/Rb IgG Biotin-free (Immunologic, Duiven, Netherlands) served as the secondary antibody. 3,3’-diaminobenzidine tetrahydrochloride (DAB, D5905, Sigma-Aldrich, St. Louis, MO, USA) was used as a substrate-staining chromogen; counterstaining was performed with Mayer’s hematoxylin (MHS32-1L, Sigma-Aldrich, St. Louis, MO, USA) [67].

The intensity of immunoreaction was measured both by determining the percentage of cells with a positive response and by the quantitative comparison of mean pixel intensity in the photomicrographs, which were firstly converted into negatives and then into 8-bit gray-scale digital images, with a scale from 0 (white pixel) to 255 (black pixel), where the higher the pixel value, the higher the intensity of the immunohistochemical reaction [26,67]. The intensity of the immunoreaction in each of the analyzed images was measured in six randomly selected areas of the positive signal. The analyses were done blindly using ImageJ software [66].

### 4.4. Western Blot

Bones stored at −80 °C were removed from the freezer and placed into a mortar and pestle kept on dry ice. The bones were then homogenized in lysis buffer (125 mM TRIS-HCl pH 6.8; 4% SDS; 10% glycerol; 100 mM DTT), boiled in a water bath for 10 min, and centrifuged at 13000× *g* for 15 min. The supernatant was then removed into new Eppendorf tubes and the pellet discarded. The Bradford method (Bradford, 1976) was employed to determine the protein content. Samples containing 80 µg of protein were separated by 12% SDS-PAGE and then electroblotted onto an Immobilon P membrane (Sigma-Aldrich, St. Louis, MO, USA). After the transfer, the membranes were blocked with 3% low fat milk in PBS for 1 h and incubated overnight with primary antibodies. Rabbit polyclonal to vascular endothelial growth factor (VEGF, Biorbyt, Wuhan, China) antibody, as well as the same MMP-8 and TIMP-2 antibodies as for immunohistochemistry, were used. The membranes were washed three times for 10 min with PBS containing 0.05% TRITON X-100 (Sigma-Aldrich, St. Louis, MO, USA) and incubated for 2 h in the presence of a 1:30,000 dilution of alkaline phosphatase-conjugated goat anti-rabbit IgG (Abcam, Cambridge, UK). The membranes were visualized after addition of BCIP (5-bromo-4-chloro-3indolyl phosphate, Sigma-Aldrich, St. Louis, MO, USA) and NBT (nitrotetrazolium blue chloride, Sigma-Aldrich, St. Louis, MO, USA), which give a blue reaction color. An anti-β-actin antibody (1:2000, Sigma-Aldrich, St. Louis, MO, USA) was used as the loading control. The blots were densitometrically quantified and normalized to their corresponding β-actin bands. The quantitative analysis of protein bands was performed using the public domain ImageJ program with gel analysis [66]. Three independent experiments were performed.

### 4.5. Statistical Analysis

The statistical analysis was performed using Statistica13.1 (Palo Alto, CA, USA) and Origin2021b (OriginLab, Northampton, MA, USA). The dataset was checked for the distribution normality by the Shapiro–Wilk test, while the homogeneity of the variance was studied using the Levene test. A mixed ANOVA, with the fumonisin treatment as a fixed effect and the animal as a random effect, was used to assess the changes under the influence of fumonisin administration. Non-parametric data were analyzed using a Kruskal–Wallis H test and the post hoc analysis used the Dunn’s test. Post hoc tests were applied (Tukey) to evaluate differences between the control and supplemented groups in terms of the analyzed parameters. Additionally, linear and quadratic trends were investigated to estimate the possible direction of observed changes. For all tests, a *p*-value < 0.05 was established as statistically significant. All the data are reported as mean ± standard error. Analysis was performed separately for each sex.

## 5. Conclusions

Our interpretation of the findings of the present study are that the toxic FB effects observed were due to FB crossing the placental barrier, affecting chondrocyte lipid metabolism and sphingosine kinase and serine/threonine-protein kinase, with resultant effects on MMPs, VEGF and TIMP. Exposure of pregnant dams to FB negatively affected the health of newborns in a dose- and sex-dependent manner, affecting bone development, offspring locomotor function and survival.

## Figures and Tables

**Table 1:** The trabecular bone histomorphometry of rat offspring following maternal exposure to 0, 60 and 90 mg/kg b.w. of fumonisins.

Dependent Variable	Sex	FB (mg/kg b.w.)			*p*-Value	*p*-Level	
		0	60	90		Linear	Quadratic
BV/TV	F	20.14 ± 1.32 ^a^	13.52 ± 0.49 ^b^	15.85 ± 0.99 ^b^	0.001	0.002	<0.001
Tb.Th (mm)		17.14 ± 0.74 ^a^	14.92 ± 0.43 ^b^	13.11 ± 0.55 ^b^	0.001	<0.001	0.002
Tb.Th_max_ (mm)		44.42 ± 2.95 ^a^	31.96 ± 0.87 ^b^	29.80 ± 1.92 ^b^	<0.001	<0.001	<0.001
Tb.Sp (mm)		74.97 ± 5.07 ^a^	99.67 ± 7.63 ^b^	85.73 ± 3.54 ^a,b^	0.025	0.085	0.011
Tb.Sp_max_ (mm)		213.83 ± 22.11	206.33 ± 15.25	174.17 ± 13.39	0.259	0.159	0.457
Tb.N		11.73 ± 0.43 ^a^	9.06 ± 0.15 ^b^	12.12 ± 0.71 ^a^	0.001	0.735	0.011
Length (mm)		10.97 ± 0.07 ^a^	9.73 ± 0.19 ^b^	9.68 ± 0.15 ^b^	<0.001	<0.001	<0.001
BV/TV	M	17.53 ± 1.03	17.20 ± 0.70	18.24 ± 0.48	0.634	0.626	0.972
Tb.Th (mm)		13.06 ± 0.26	13.67 ± 0.62	14.70 ± 0.72	0.156	0.071	0.223
Tb.Th_max_ (mm)		29.70 ± 2.93	35.84 ± 3.08	34.24 ± 1.55	0.257	0.171	0.105
Tb.Sp (mm)		68.76 ± 3.85 ^a^	87.67 ± 6.85 ^b^	75.00 ± 2.85 ^a,b^	0.041	0.181	0.024
Tb.Sp_max_ (mm)		151.17 ± 11.91 ^a^	242.67 ± 16.52 ^b^	179.50 ± 14.01 ^a^	0.001	0.062	<0.001
Tb.N		13.45 ± 0.84	12.68 ± 0.69	12.49 ± 0.36	0.567	0.299	0.339
Length (mm)		11.54 ± 0.09 ^a^	10.76 ± 0.28 ^b^	10.04 ± 0.25 ^b^	0.001	<0.001	0.003

Results are presented as mean ± SE with corresponding statistical analysis. FB—fumonisin, BV/TV—bone volume over the total volume, Tb.Th—trabecular thickness, Tb.Th_max_—maximum trabecular thickness, Tb.Sp—trabecular space, Tb.Sp_max_—maximum trabecular space, Tb.N—trabecular number, F—female, M—male. Statistically significant differences between groups (at *p*-value < 0.05) are indicated by ^a^ and ^b^.

**Table 2:** The intensity of MMP-13, MMP-8 and TIMP-2 immunoreaction in bone and articular tissue of female rat offspring following maternal exposure to 0, 60 and 90 mg/kg b.w. of fumonisins.

Dependent Variable	Region		FB (mg/kg b.w.)			*p*-Value	*p*-Level	
			0	60	90		Linear	Quadratic
MMP-13	growing plate	proliferative	116.66 ± 7.79 ^a^	78.09 ± 1.87 ^b^	98.92 ± 8.76 ^a,b^	<0.001	0.026	0.002
		hypertrophic	86.66 ± 1.86 ^a^	111.41 ± 7.38 ^b^	119.13 ± 7.43 ^b^	0.005	0.002	0.003
	articular		150.76 ± 12.45 ^a^	101.29 ± 3.27 ^b^	86.97 ± 2.61 ^b^	<0.001	<0.001	<0.001
	trabecular	osteocyte	190.59 ± 9.27 ^a^	158.14 ± 7.42 ^b^	139.62 ± 6.72 ^b^	0.001	<0.001	0.002
		matrix	158.23 ± 7.04 ^a^	112.42 ± 4.86 ^b^	92.23 ± 7.72 ^b^	<0.001	<0.001	<0.001
	compact	osteocyte	184.21 ± 10.01 ^a^	210.61 ± 5.77 ^a^	116.11 ± 5.56 ^b^	<0.001	<0.001	0.065
		matrix	134.83 ± 8.40	152.05 ± 4.21	120.60 ± 1.42	0.054	0.282	0.253
MMP-8	growing plate	proliferative	93.27 ± 1.05 ^a^	80.76 ± 3.91 ^b^	77.28 ± 2.31 ^b^	0.002	0.001	0.001
		hypertrophic	125.69 ± 11.56	105.63 ± 4.74	104.15 ± 9.98	0.188	0.078	0.092
	articular		191.28 ± 11.99 ^a^	117.59 ± 8.28 ^b^	111.88 ± 5.04 ^b^	<0.001	<0.001	<0.001
	trabecular	osteocyte	220.02 ± 12.91 ^a^	178.91 ± 4.73 ^b^	105.58 ± 9.40 ^c^	<0.001	<0.001	<0.001
		matrix	139.81 ± 3.09 ^a^	99.48 ± 10.87 ^b^	151.86 ± 14.32 ^a^	0.009	0.912	0.002
	compact	osteocyte	174.57 ± 9.16 ^a^	220.66 ± 3.99 ^b^	128.16 ± 7.34 ^c^	<0.001	0.009	0.045
		matrix	125.95 ± 6.99	110.64 ± 4.06	118.98 ± 3.38	0.154	0.230	0.070
TIMP-2	growing plate	proliferative	242.35 ± 3.18 ^a^	217.17 ± 5.67 ^b^	88.21 ± 5.64 ^c^	<0.001	<0.001	<0.001
		hypertrophic	226.13 ± 3.68 ^a^	185.12 ± 6.18 ^b^	132.12 ± 5.08 ^c^	0.001	<0.001	<0.001
	articular		166.55 ± 6.25	182.75 ± 2.57	193.23 ± 15.33	0.180	0.069	0.137
	trabecular	osteocyte	221.86 ± 5.93	205.72 ± 3.31	225.94 ± 1.84	0.003	0.994	0.039
		matrix	151.75 ± 3.68	136.24 ± 0.97	139.78 ± 7.48	0.092	0.062	0.061
	compact	osteocyte	237.74 ± 3.68 ^a^	180.51 ± 1.86 ^b^	198.87 ± 3.99 ^c^	<0.001	<0.001	<0.001
		matrix	156.21 ± 6.70	140.79 ± 2.21	141.25 ± 5.25	0.081	0.055	0.221

Results are presented as mean ± SE with corresponding statistical analysis. FB—fumonisin, MMP-13—matrix metalloproteinase 13, MMP-8—matrix metalloproteinase 8, TIMP-2—metalloproteinase inhibitor. Statistically significant differences between groups (at *p*-value < 0.05) are indicated by ^a^, ^b^ and ^c^.

**Table 3:** The intensity of MMP-13, MMP-8 and TIMP-2 immunoreaction in bone and articular tissue of male rat offspring following maternal exposure to 0, 60 and 90 mg/kg b.w. of fumonisins.

Dependent Variable	Region		FB (mg/kg b.w.)			*p*-Value	*p*-Level	
			0	60	90		Linear	Quadratic
MMP-13	growing plate	proliferative	89.99 ± 3.51	87.71 ± 1.93	94.59 ± 11.12	0.771	0.719	0.972
		hypertrophic	155.85 ± 13.50	116.79 ± 10.17	141.59 ± 9.09	0.071	0.201	0.057
	articular		124.54 ± 8.22 ^a^	81.79 ± 1.59 ^b^	83.79 ± 3.25 ^b^	<0.001	<0.001	<0.001
	trabecular	osteocyte	196.98 ± 4.73 ^a^	156.76 ± 4.04 ^b^	117.00 ± 1.33 ^c^	<0.001	<0.001	0.960
		matrix	168.63 ± 8.26 ^a^	106.15 ± 9.95 ^b^	111.16 ± 4.21 ^b^	<0.001	<0.001	<0.001
	compact	osteocyte	202.95 ± 8.29 ^a^	130.40 ± 12.72 ^b^	128.04 ± 3.67 ^b^	<0.001	<0.001	<0.001
		matrix	121.41 ± 5.59 ^a^	158.13 ± 4.11 ^b^	110.34 ± 2.70 ^a^	<0.001	0.789	0.001
MMP-8	growing plate	proliferative	113.24 ± 4.13	123.66 ± 1.33	126.96 ± 8.85	0.237	0.097	0.134
		hypertrophic	201.27 ± 12.25	208.31 ± 5.99	172.15 ± 19.43	0.176	0.237	0.852
	articular		181.91 ± 9.48 ^a^	118.87 ± 8.88 ^b^	133.31 ± 6.81 ^b^	<0.001	<0.001	<0.001
	trabecular	osteocyte	222.45 ± 3.89 ^a^	175.96 ± 8.45 ^b^	149.31 ± 1.68 ^c^	<0.001	<0.001	<0.001
		matrix	104.90 ± 5.67	123.29 ± 9.80	131.28 ± 9.49	0.114	0.048	0.074
	compact	osteocyte	206.23 ± 5.28 ^a^	117.86 ± 7.97 ^b^	208.96 ± 7.25 ^a^	<0.001	0.105	<0.001
		matrix	130.26 ± 7.47 ^a^	88.12 ± 5.28 ^b^	180.73 ± 6.57 ^c^	<0.001	0.002	0.075
TIMP-2	growing plate	proliferative	246.78 ± 2.59 ^a^	168.37 ± 10.77 ^b^	169.25 ± 3.73 ^b^	<0.001	<0.001	<0.001
		hypertrophic	243.00 ± 2.21 ^a^	94.88 ± 6.88 ^b^	146.66 ± 2.10 ^c^	<0.001	<0.001	<0.001
	articular		196.23 ± 2.65 ^a^	147.51 ± 11.60 ^b^	175.32 ± 10.45 ^a,b^	0.007	0.041	0.003
	trabecular	osteocyte	243.27 ± 1.94 ^a^	222.82 ± 5.58 ^b^	158.03 ± 4.52 ^c^	<0.001	<0.001	<0.001
		matrix	153.18 ± 3.38 ^a^	136.16 ± 1.62 ^b^	110.27 ± 2.42 ^c^	<0.001	<0.001	<0.001
	compact	osteocyte	193.98 ± 4.30	189.69 ± 5.23	193.03 ± 10.20	0.904	0.862	0.714
		matrix	124.61 ± 5.50	125.30 ± 2.99	131.35 ± 8.04	0.679	0.480	0.749

Results are presented as mean ± SE with corresponding statistical analysis. FB—fumonisin, MMP-13—matrix metalloproteinase 13, MMP-8—matrix metalloproteinase 8, TIMP-2—metalloproteinase inhibitor 2. Statistically significant differences between groups (at *p*-value < 0.05) are indicated by ^a^, ^b^ and ^c^.

**Table 4:** Western blot protein expression. The relative abundance of MMP-8, TIMP-2 and VEGF was evaluated densitometrically and expressed as the ratio relative to β-actin used as a loading control across all replicates as described in the Materials and Methods section.

Dependent Variable	Sex	FB (mg/kg b.w.)			*p*-Value	*p*-Level	
		0	60	90		Linear	Quadratic
MMP-8	F	1.096 ± 0.022 ^a^	1.303 ± 0.041 ^a^	0.886 ± 0.086 ^b^	<0.001	0.103	0.235
TIMP-2		0.655 ± 0.012 ^a,b^	0.803 ± 0.055 ^a^	0.486 ± 0.063 ^b^	0.001	0.116	0.366
VEGF		0.992 ± 0.058 ^a^	1.077 ± 0.019 ^a,b^	1.148 ± 0.036 ^b^	0.005	0.002	0.010
MMP-8	M	1.070 ± 0.078 ^a^	1.110 ± 0.009 ^a^	0.783 ± 0.055 ^b^	<0.001	0.067	0.455
TIMP-2		1.053 ± 0.140	1.223 ± 0.085	0.860 ± 0.073	0.076	0.384	0.619
VEGF		0.804 ± 0.028 ^a^	1.067 ± 0.049 ^b^	0.889 ± 0.051 ^a^	0.002	0.051	0.002

Data are presented as mean ± SE with corresponding statistical analysis. FB—fumonisin, MMP-8—matrix metalloproteinase 8, TIMP-2—metalloproteinase inhibitor 2, VEGF—vascular endothelial growth factor A, F—female, M—male. Statistically significant differences between groups (at *p*-value < 0.05) are indicated by ^a^ and ^b^.

## Data Availability

The data presented in this study are available on request from the corresponding author.

## Supplementary Materials

The following are available online at https://www.mdpi.com/article/10.3390/ijms222212528/s1.
