# Supplementary material for: Trabecular Bone Parameters, TIMP-2, MMP-8, MMP-13, VEGF Expression and Immunolocalization in Bone and Cartilage in Newborn Offspring Prenatally Exposed to Fumonisins
Source: Int J Mol Sci. 2021 Nov 20;22(22):12528. doi: 10.3390/ijms222212528 (PMC8623786; doi:10.3390/ijms222212528)
Supplement: Supplementary file 1 [file ijms-22-12528-s001.zip › ijms-1462471-supplementary.pdf]

SUPPLEMENTARY MATERIAL

Trabecular Bone Parameters, TIMP-2, MMP-8, MMP-13, VEGF Expression and Immunolocalization in Bone and Cartilage in Newborn Offspring Prenatally Exposed to Fumonisins

Ewa Tomaszewska [1,*], Halyna Rudyk [2], Izabela Świetlicka [3,*], Monika Hułas-Stasiak [4], Janine Donaldson [5], Marta Arczewska [3], Siemowit Muszyński [3], Piotr Dobrowolski [4], Maria Mielnik-Błaszczak [6], Marcin B. Arciszewski [7], Volodymyr Kushnir [2], Oksana Brezvyn [2], Viktor Muzyka [2], and Ihor Kotsyumbas [2]

[1] Department of Animal Physiology, Faculty of Veterinary Medicine, University of Life Sciences in Lublin, Akademicka St. 12, 20-950 Lublin, Poland; ewaRST@interia.pl (E.T.)
[2] State Scientific Research Control Institute of Veterinary Medicinal Products and Feed Additives, Donetska St. 11, 79000 Lviv, Ukraine; galusik.77@gmail.com (H.R.); wolodjak@gmail.com (V.K.); brezvun@gmail.com (O.B.); muzyka@scivp.lviv.ua (V.M.); dir@scivp.lviv.ua (I.K.)
[3] Department of Biophysics, Faculty of Environmental Biology, University of Life Sciences in Lublin, Akademicka St. 13, 20-950 Lublin, Poland; izabela.swietlicka@up.lublin.pl (I.Ś.); marta.arczewska@up.lublin.pl (M.A.); siemowit.muszynski@up.lublin.pl (S.M.)
[4] Department of Functional Anatomy and Cytobiology, Faculty of Biology and Biotechnology, Maria Curie-Sklodowska University, 19 Akademicka St., 20-033 Lublin, Poland; piotr.dobrowolski@umcs.lublin.pl (P.D.)
[5] School of Physiology, Faculty of Health Sciences, University of the Witwatersrand, 7 York Road, Parktown, Johannesburg, 2193, South Africa; janine.donaldson@wits.ac.za (J.D.)
[6] Chair and Department of Developmental Dentistry, Medical University of Lublin, 7 Karmelicka St., 20-081 Lublin, Poland; maria.mielnik-blaszczak@umlub.pl (MM-B)
[7] Department of Animal Anatomy and Histology, University of Life Sciences in Lublin, 20-950 Lublin, Poland; mb.arciszewski@wp.pl (M.B.A.)

* Correspondence: ewaRST@interia.pl (E.T.); izabela.swietlicka@up.lublin.pl (I. Ś.)

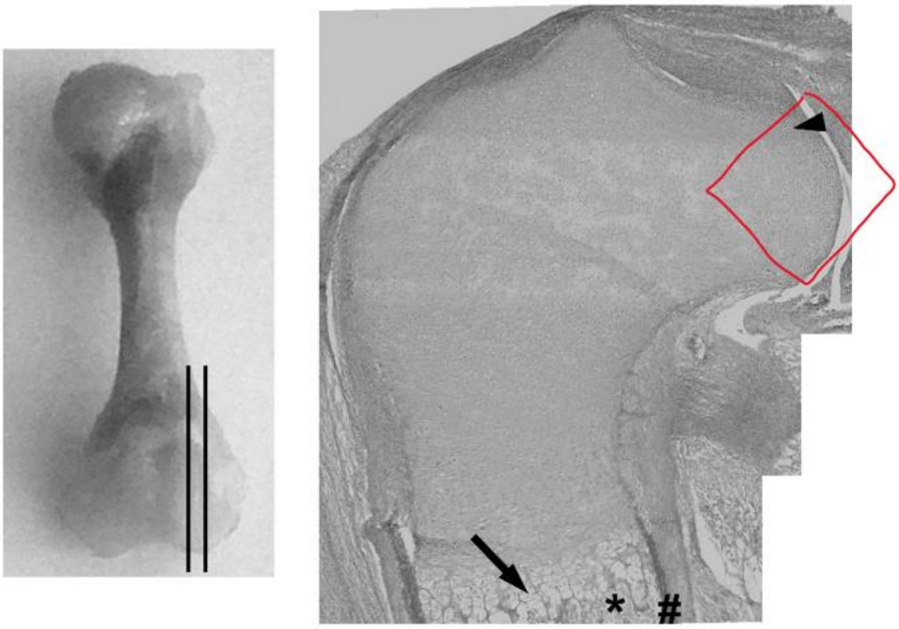

Figure S1. The location selection and the size of areas of interest analyzed in femoral distal epiphysis and sagittal section, chosen based on motoric properties of rat body and the knee joint. The site of femoral sections is marked by black parallel lines. The area of articular cartilage of interest analyzed is marked by the red square. Cartilage and trabeculae analysis. Arrowhead – articular cartilage, arrow – growth plate, * – trabecular bone, # - Compact bone.

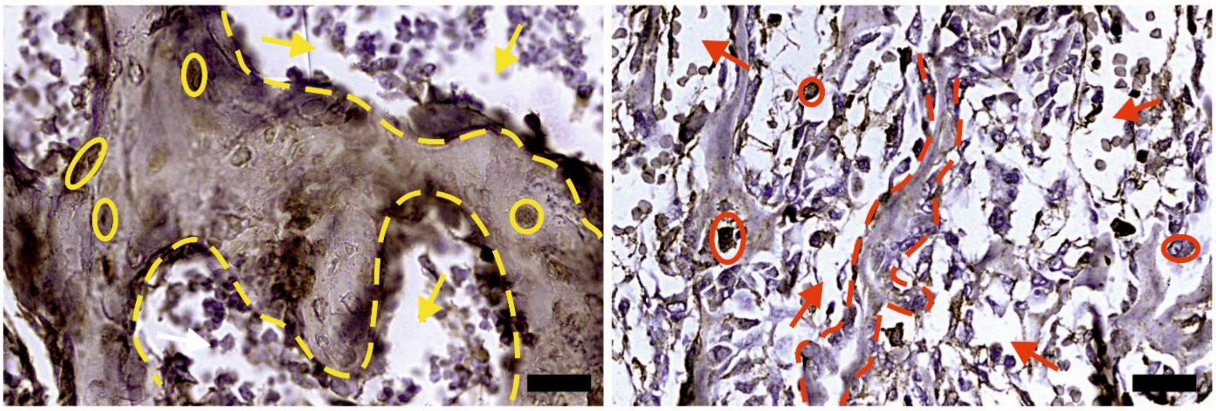

Figure S2. Representative images of the immunohistochemical reactions from the compact bone of newborn rats with signed trabeculae (dashed lines), osteocytes (ovals) and marrow space (arrows). All the scale bars represent 40 μm

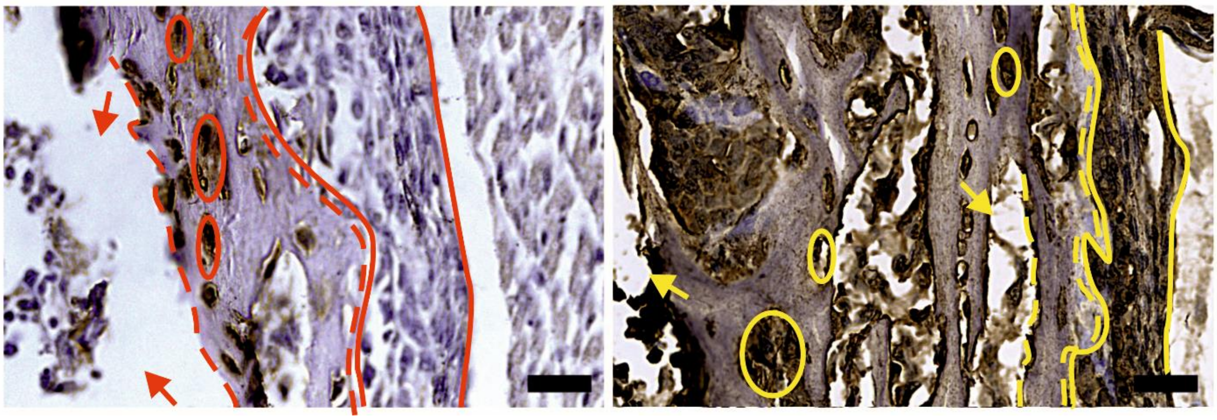

Figure S3. Representative images of the immunohistochemical reactions from the trabecular bone of newborn rats with signed periosteum (solid line), compact bone (dashed lines), osteocytes (ovals) and bone marrow cavity (arrows). All the scale bars represent 40 μm

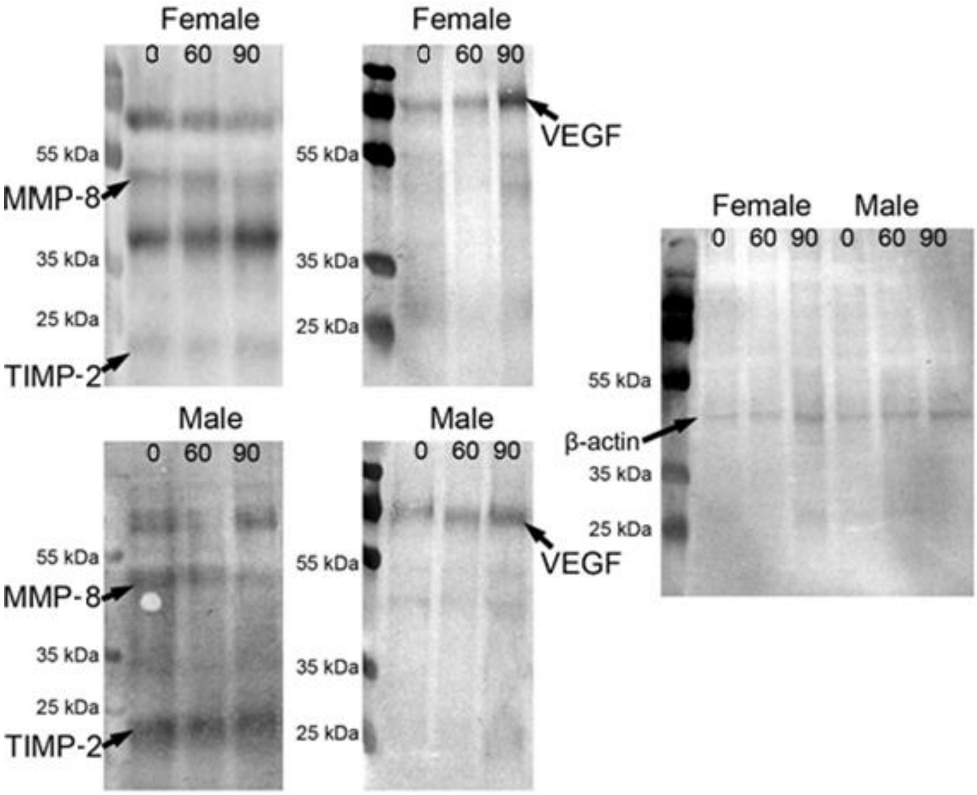

Figure S4. Representative, original Western blot membranes presenting the level: tissue inhibitor of metalloproteinases 2 (TIMP-2, 23 kDa), matrix metalloproteinase 8 (MMP-8, 53 kDa) and vascular endothelial growth factor (VEGF, 68-70 kDa) as well as β-actin as representative loading control. For MMP-8, the 68 kDa band as well as 38 kDa band (observed in female rats) appear to be other glycosylated forms of MMP-8 that can be recognized by the monoclonal antibodies used in this study, however, according to the manufacturer's datasheet, only the 53 kDa band was quantified.

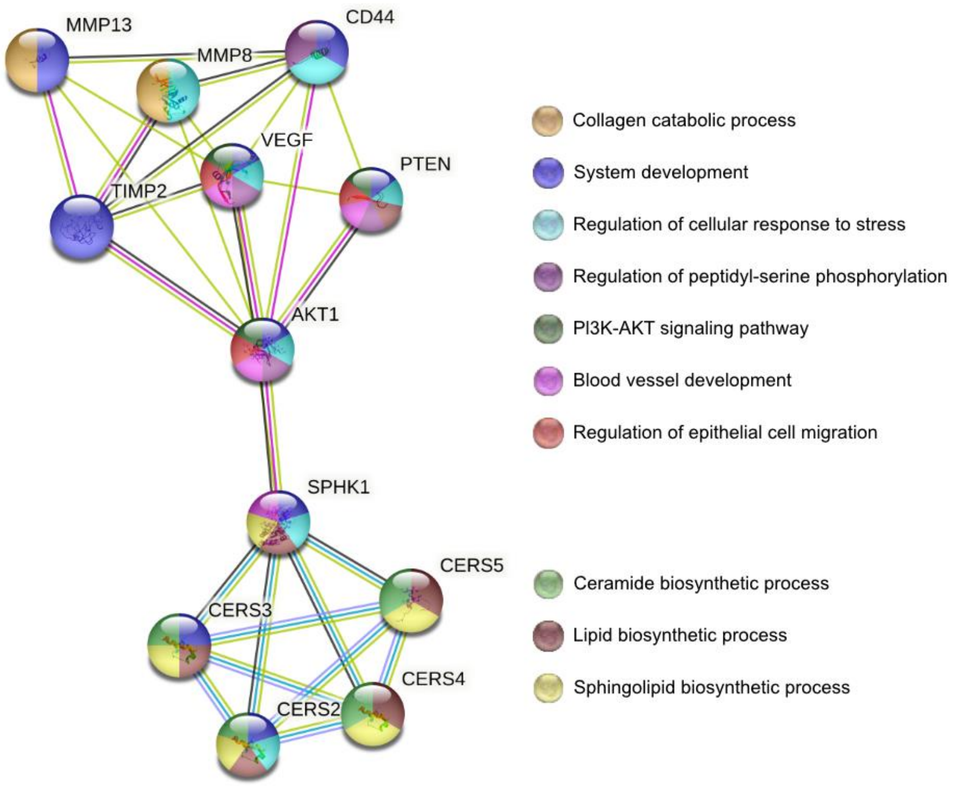

Figure S5. STRING protein-protein interaction diagram between examined proteins (MMP-8, MMP-13, TIMP-2, VEGF) and lipid biosynthesis-related proteins inhibited by FB (ceramide synthase and serine/threonine phosphatase). Gene ontology pathway analysis revealed that presented proteins are involved in biological processes of system development, regulation of cellular response to stress, lipid biosynthesis process, sphingolipid biosynthesis process, ceramide biosynthesis process, regulation of peptidyl-serine phosphorylation, blood vessel development, regulation of epithelial cell migration-orange, collagen catabolic process, PI3K-Akt signaling pathway; https://string-db.org/, accessed on 12.11.2021.

CD44 - Receptor for hyaluronic acid. Mediates cell-cell and cell-matrix interactions through its affinity for HA, and possibly also through its affinity for other ligands such as osteopontin, collagens, and matrix metalloproteinases (MMPs).
MMP - matrix metalloproteinase
TIMP - tissue inhibitor of metalloproteinases
VEGF - vascular endothelial growth factor
PTEN- a serine/threonine protein phosphatase
AKT1- serine/threonine- protein kinase
SPHK1 - Sphingosine kinase; Catalyzes the phosphorylation of sphingosine to form sphingosine 1-phosphate (SPP), a lipid mediator with both intra- and extracellular functions
CERS- Ceramide synthase (CERS5- Inhibited by fumonisin B1)
